# How reliable are Chinese hamster ovary (CHO) cell genome‐scale metabolic models?

**DOI:** 10.1002/bit.28366

**Published:** 2023-03-18

**Authors:** Benjamin Strain, James Morrissey, Athanasios Antonakoudis, Cleo Kontoravdi

**Affiliations:** ^1^ Department of Chemical Engineering Imperial College London London UK

**Keywords:** bioprocessing, Chinese hamster ovary cells, flux analysis, metabolic network, recombinant protein production

## Abstract

Genome‐scale metabolic models (GEMs) possess the power to revolutionize bioprocess and cell line engineering workflows thanks to their ability to predict and understand whole‐cell metabolism in silico. Despite this potential, it is currently unclear how accurately GEMs can capture both intracellular metabolic states and extracellular phenotypes. Here, we investigate this knowledge gap to determine the reliability of current Chinese hamster ovary (CHO) cell metabolic models. We introduce a new GEM, iCHO2441, and create CHO‐S and CHO‐K1 specific GEMs. These are compared against iCHO1766, iCHO2048, and iCHO2291. Model predictions are assessed via comparison with experimentally measured growth rates, gene essentialities, amino acid auxotrophies, and ^13^C intracellular reaction rates. Our results highlight that all CHO cell models are able to capture extracellular phenotypes and intracellular fluxes, with the updated GEM outperforming the original CHO cell GEM. Cell line‐specific models were able to better capture extracellular phenotypes but failed to improve intracellular reaction rate predictions in this case. Ultimately, this work provides an updated CHO cell GEM to the community and lays a foundation for the development and assessment of next‐generation flux analysis techniques, highlighting areas for model improvements.

## Graphical abstract

In this work, Strain et al present a new consolidated Chinese Hamster Ovary (CHO) cell Genome‐scale Model (GEM), iCHO2441. They assess the predictive performance of this model against previous CHO cell GEMs by comparison with experimentally measured growth rates, gene essentialities, amino acid auxotrophies, and 13C intracellular reaction rates. Their work demonstrates the strengths and weaknesses of CHO cell GEMs, highlighting areas for model improvements, and demonstrating these models are reliable enough to be embedded in industrial workflows.

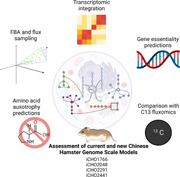

## 1 INTRODUCTION

Chinese hamster ovary (CHO) cells are the leading platform for the production of many therapeutic recombinant proteins (Walsh, 2018). The increasing demand for recombinant therapeutic proteins highlights the need to improve the efficiency and yield of these biopharmaceutical products from mammalian cells, which is achievable through an in‐depth understanding of cellular functioning. Metabolic modeling has emerged both as a prediction tool for the engineering of CHO cells and as a theoretical framework to elucidate fundamental biological questions (Rejc et al., 2017).

Since the publication of the first Chinese hamster genome‐scale model (GEM) in 2016, iCHO1766 (Hefzi et al., 2016), there has been an acceleration of advancements and applications in the field of CHO cell genome‐scale modeling. These include using CHO cell metabolic models as a tool to predict amino acid uptake/secretion to assist process control (Schinn et al., 2021a), identifying burdensome host cell protein for knockout (Kol et al., 2020), predicting product glycosylation (Antonakoudis et al., 2021), and designing feeding strategies to optimize monoclonal antibody production (Fouladiha et al., 2020).

Despite the promise of these applications, there are several fundamental challenges with CHO cell GEMs and the flux analysis methods used to solve them. Full‐scale CHO cell GEMs are large (>6000 reactions) and the data used to inform them are limited, which results in an underdetermined system of equations. The solution of GEMs relies on constraint‐based methods, such as flux balance analysis (FBA), to predict steady‐state flux distributions. There are typically an infinite number of possible intracellular flux distributions to satisfy a given optimum and intracellular reaction fluxes can vary within a large range for a given solution. The underdetermined nature of GEM analysis poses limitations for the application of in silico modeling techniques for CHO cell systems as accurate predictions of intracellular fluxes are essential in the rational design of new engineering strategies.

While some attempts have been made to measure CHO cell GEM predictive performance (Schinn et al., 2021b; Széliová, 2021a, 2021b), the effectiveness of CHO cell GEMs at capturing both intracellular flux distributions and extracellular phenotypes has not been comprehensively assessed. In this paper we present an evaluation of extracellular and intracellular predictive capabilities of CHO cell GEMs, using a holistic ^13^C‐labeled fluxomic assessment methodology. We constrain the GEMs with published metabolomic datasets and employ FBA to assess a variety of experimentally validated extracellular traits including gene essentiality, growth rate, and amino acid auxotrophy predictions. We then employ flux sampling to assess the feasible solution space of intracellular flux predictions and compare against the experimentally determined ^13^C‐labeled reaction rates to evaluate the accuracy of intracellular flux predictions of CHO cell GEMs. We introduce an updated GEM, iCHO2441, and compare this against previous published CHO cell GEMs (iCHO1766, iCHO2048, iCHO2291), as well as CHO‐S and CHO‐K1 specific models. We further evaluate the reliability of CHO cell GEMs, with the findings supporting the application of GEMs in industrial settings.

## 2 METHODS

### 2.1 Experimental data set

#### 2.1.1 ^13^C‐labeled metabolomics data set

The experimental data set used herein contains 31 measurements, from eight published studies as detailed in Table 1. Each measurement comprises a set of extracellular metabolite uptake/secretion rates, growth rates, and intracellular reactions and is available in the Supporting Information.

**Table 1:** C^13^‐labeled experiments used for flux analysis and model evaluation.

Cell line and process description	Producer/non‐ producer	Cell culture stage	Number of measurements	Reference
CHO‐S across several stages of fed‐batch culture	Producer	Early exponential, late exponential, stationary and decline	4	Templeton et al. (2013)
CHO‐S with varying expressions of antiapoptotic BCL2 gene	Non‐producer	Early and late exponential	6	Templeton et al. (2014)
CHO‐S with/without expression of antiapoptotic BCL2 gene	Both	Stationary	9	Templeton, Smith, et al. (2017)
CHO‐S in fed‐batch and perfusion	Producer	Perfusion and stationary	2	Templeton, Xu, et al. (2017)
CHO‐S with/without low ammonium feeds and supplementation	Producer	Late exponential	3	McAtee Pereira et al. (2018)
CHO–Cum2 cells (expression of reverse cumate transactivator) with/without induction of antibody expression	Producer	Late exponential	2	Sheikholeslami et al. (2013)
Induced CHO‐Cum2 cells with high/low glutamine feeding	Producer	Late exponential	2	Sheikholeslami et al. (2014)
CHO‐K1	Non‐producer	Late exponential	1	Nicolae et al. (2014)

#### 2.1.2 Data processing

^13^C‐labeled reactions from each study were compiled and adjusted to comply with the set of ^13^C reactions used in the master data set. Some reactions did not have labeled fluxes for every experiment and reactions that had data in less than 40% of experiments were not proceeded for further analysis. This resulted in a final list of 49 intracellular reactions. Metabolite uptake rates and ^13^C fluxes were converted into units of mmol gDCW^−1^ h^−1^ using the dry cell weight measured experimentally for each cell line as reported in the associated publication. The amino acid (AA) composition of the recombinant protein products was converted to units of mol AA per mol product.

#### 2.1.3 ^13^C to model reaction mapping

To compare CHO cell model predictions with experimental flux data, the ^13^C‐labeled reactions were mapped to the reactions in our metabolic models. The draft mapping was modified from Széliová, Štor, et al. (2020) and is available in Supporting Information: Table S4. One‐to‐one mapping was not possible for most reactions as the ^13^C‐labeling lumped several reactions into one. The net flux through the lumped reactions was calculated by considering the model reactions as an “electric circuit” by splitting the model reactions into “parallel” and “series” type connections. Reactions in parallel were first summed and then treated as a reaction in series, and then the minimum of all reactions in series was taken to find the overall flux. The following additional rules were applied:

- The directionality of models was considered by multiplying the predicted flux by −1 if the model reaction ran in the opposite direction.
- The stoichiometry of models was considered by multiplying the predicted flux by the correct integer to match with ^13^C‐labeled stoichiometry.
- In case of multiple equivalent reactions occurring in several compartments, their individual contributions were summed up.

### 2.2 Creation of updated CHO GEM, iCHO2441

iCHO1766 was obtained from the Bigg models database (King et al., 2016), while iCHO2291 was obtained via the BioModels database (Malik‐Sheriff et al., 2020). The expanded iCHO2441 GEM was constructed by coupling the secretory machinery presented in Gutierrez et al. (2020) to the recently published updated iCHO2291 (Yeo et al., 2020). This was achieved by adapting the Jupyter Notebooks developed by Gutierrez et al. (2020) to use the updated iCHO2291 as a base model to which secretory reactions may be added. In brief, information of each secreted product, including amino acid composition, presence of a signal peptide, number of disulfide bonds, number of core N‐linked glycans, and molecular weight, were fed to the notebook and used to add the appropriate secretory pathway reactions to the model (Supporting Information: Table S1). For intracellular flux prediction, a custom model was generated for each secreted product using product composition data. iCHO2048 was constructed in an identical manner using iCHO1766 as a base model. For auxotrophy and gene essentiality predictions, a generic IgG structure was used to add secretory reactions to the model (Supporting Information: Table S1). A generic form of iCHO2441 is available for download from BioModels database (Malik‐Sheriff et al., 2020) with identifier MODEL2206100001.

### 2.3 Creation of cell line‐specific GEMs

#### 2.3.1 Transcriptomics datasets

For this work, two transcriptomics datasets were utilized to generate CHO‐K1 and CHO‐S cell line‐specific models. For CHO‐S, RNA‐Seq data were obtained from existing work (Hefzi et al., 2016). Briefly, CHO‐S cells were grown in CD‐CHO medium (Life Technologies # 10743‐029) supplemented with 8 mM l‐glutamine (Life Technologies # 25030‐024) at 37°C. RNA was extracted at 10 time intervals through culture. RNA libraries for sequencing were prepared using TruSeq Stranded mRNA Sample prep (Illumina). Libraries were clustered using cBot and sequenced on HiSeq. 2500 (HiSeq Control Software 2.2.38/RTA 1.18.61) with a 2 × 101 setup in RapidHighOutput mode. Bcl to Fastq conversion was performed using bcl2Fastq v1.8.3 from the CASAVA software suite. Sequencing was carried out using Illumina GA2 technology with paired‐end reads, where raw counts were converted to fragments per kilobase million (FPKM).

For CHO‐K1, RNA‐Seq data were obtained from Sumit et al. (2019). In brief, CHO‐K1 cells were grown in modified CD‐CHO medium at 37°C. RNA was extracted at 6 time intervals through culture. Samples were run on Illumina HiSeq. 2000 (Illumina). RNA‐Seq data were mapped to CHO genome using subjunc aligner program from subread1.4.6 package. The alignment bam files were compared against the gene annotation GFF file, and raw counts for each gene were generated using the featureCounts tool, where raw counts were converted to reads per kilobase per million (RPKM).

For input into model reduction algorithms, RNA‐Seq data from both CHO‐S and CHO‐K1 datasets were averaged. For mapping to reactions, gene IDs in the CHO‐K1 data set were converted to entrez IDs using the BioMart (Smedley et al., 2009) package.

#### 2.3.2 GIMME

The Gene Inactivity Moderated by Metabolism and Expression (GIMME) algorithm (Becker & Palsson, 2008) was used to generate one set of cell line‐specific models. Genes were mapped to reactions within iCHO2441 using the “mapExpressionToReactions” function in the COBRA toolbox (Heirendt et al., 2019), where the minimum expression value was used for “AND” and maximum expression value was used for “OR” GPR associations. Following the approach suggested by Becker and Palsson (2008), reactions that were not mapped to gene expression data were given a score of −1 to favor their inclusion in the final model. iCHO2441 was first constrained per experiment with experimental uptake rates (Supporting Information: Table S1) before reconstructions were generated using the GIMME function within the CORBA toolbox. For IgG‐producing cell lines, the “biomass_cho_producing” objective function was used. For nonproducing cell lines, the “biomass_cho” objective function was used. In all instances, the optimality threshold was set to 0.9 and the experimental biomass constraint was set only as a lower bound. In line with the methodology utilized to produce current CHO cell line‐specific GEMs (Hefzi et al., 2016; Yusufi et al., 2017), genes were called as present if their RPKM/FPKM was greater than 1.

#### 2.3.3 CORDA coupled to zFPKM/zRPKM transformation

A second set of cell line‐specific GEMs was generated using a novel application of the Cost Optimization Reaction Dependency Assessment (CORDA) algorithm (Schultz & Qutub, 2016). In brief, RNA‐Seq values were z‐transformed following the approach developed by Hart et al. (2013) to zFPKM/zRPKM values, using the function within Bioconductor for R 4.1.0 following the equation:

$$\text{zFPKM}=\frac{\log2(\text{FPKM})-\mu }{\sigma },$$

where μ is the log2‐transformed FPKM value at the maximum value of the density plot and σ is the SD.

As per the recommendation by Hart et al., genes with transformed values less than −3 were classed as “not expressed/negative confidence” (class −1). Genes with transformed values between −3 and −1.5 were classed as “low confidence expressed” (class 1). Genes with transformed values between −1.5 and 0 were classed as “medium confidence expressed” (class 2). Genes with transformed values greater than 0 were classed as “expressed/high confidence” (class 3). These classifications were mapped to reactions within iCHO2441 using the method described above, where unclassified reactions were given a class of 0. Reconstructions were generated per experiment using the CORDA function for python which took iCHO2441 constrained with experimental uptakes (Supporting Information: Table S2) as a base model and the gene confidence classifications as inputs. An in‐depth rationale behind this novel approach is presented in Supporting Information: Material 1.

### 2.4 Model analysis

#### 2.4.1 Flux analysis

Models were constrained using the experimentally measured uptake rates of nutrients/metabolites, growth rate, and antibody productivity within the data set (Supporting Information: Table S2). Default bounds for reversible reactions were [−1000, 1000] and [0, 1000] for irreversible reactions. To account for experimental error, the lower and upper experimental values were used as lower and upper bounds, respectively. For exchange reactions not accounted for within experimental metabolomic data, the bounds remained the same as the default values of the iCHO1766 model (Hefzi et al., 2016).

For intracellular flux predictions, the “biomass_cho_producing” reaction was used to constrain experimental growth rate. For nonproducing cell lines, the “biomass_cho” reaction was used. The rationale behind using the default biomass compositions, rather than using the experimentally determined composition (if available) or other published biomass compositions (Széliová, Ruckerbauer, et al., 2020), is that, in our opinion, the default biomass function is a key component of a genome scale model. It is rare in CHO cell applications that the biomass composition is measured and utilized in flux analysis; most instances of model application use the default composition. While experimentally determining and utilizing condition‐ and cell line‐specific biomass compositions would improve model predictions (Lakshmanan et al., 2019; Schinn et al., 2021b; Széliová et al., 2021), for the purpose of this analysis we believe the default compositions provide a more representative comparison across models.

While the biomass reactions were retained from the original model, the antibody synthesis reactions were added separately. For iCHO1766 and iCHO2291, the original amino acid composition of the “igg_g” formation reactions was modified to match the experimental amino acid composition. For iCHO2048 and iCHO2441 custom secretory reactions were added using the method described above. When the recombinant protein composition was not defined, the default “igg_g” formation values were used.

*Flux sampling*: To investigate predicted intracellular reaction fluxes, optGpSampler (Megchelenbrink et al., 2014) was utilized to uniformly sample the metabolic flux solution space of all models using the sample function in the flux analysis submodule of COBRApy (Ebrahim et al., 2013). The solution space was sampled 50,000,000 times, of which solutions were stored every 10,000 iterations, resulting in 5000 data‐points per reaction.

*FBA*: For FBA simulations of theoretical max growth rate prediction, biomass growth was used as an objective function and not constrained by the model. For producing clones “biomass_cho_producing” objective function was used and for nonproducing cell lines, the “biomass_cho” objective function was used. For FBA simulations of theoretical max antibody flux, antibody demand reactions were used as an objective function and not constrained by the model.

#### 2.4.2 Model performance evaluation

##### Pearson correlation coefficient

The agreement between model predictions and experimentally observed values is measured with several metrics. The first of these is the Pearson product–moment correlation coefficient, ρ, which is used as a statistical measurement of the correlation between the experimental reaction fluxes and the predicted fluxes. The following equation is used to calculate ρ:

$$\rho =\frac{\sum_{i=1}^{n}\left(p_{i}-{\bar{p}}_{i}\right)\left(o_{i}-{\bar{o}}_{i}\right)}{\sqrt{{\sum_{i=1}^{n}\left(p_{i}-{\bar{p}}_{i}\right)}^{2}{\sum_{i=1}^{n}\left(o_{i}-{\bar{o}}_{i}\right)}^{2}}},$$

where p is the mean predicted value, o is the median experimentally observed value, ${\bar{p}}_{i}$ is the sample mean of predicted values, and ${\bar{o}}_{i}$ is the sample mean of observed values.

##### Capability

To measure the ability of each model to accurately predict intracellular flux values a novel metric termed Capability was developed. Herein, each of the 5000 samples was mapped to the ^13^C‐labeled reactions using the method above. If this value fell within the upper and lower bounds of the ^13^C determined value a hit was recorded. The following equation was used to define a hit:

$$\mathrm{Hit}=O_{lb}\geq p\geq O_{\mathrm{ub}},$$

where p is the predicted value, $O_{\mathrm{lb}}$ is the lower experimentally observed value and $O_{\mathrm{ub}}$ is the upper experimentally observed value. This hit value was converted to a percentage Capability using the following equation:

$$\mathrm{Capability}\;\left(\%\right)=\frac{\#\mathrm{Hits}}{\#\mathrm{Total}\;\mathrm{Samples}}\times 100.$$

##### Model auxotrophy predictions

To assess if cell line‐specific GEMs could capture known CHO cell auxotrophies, exchange reactions were constrained to 0 for the respective amino acid and viability was evaluated by running FBA biomass maximization.

##### Gene essentiality analysis

To further assess the quality of GEMs, models were evaluated by comparing predicted gene essentiality with experimentally determined essential genes for a CHO‐S cell line taken from Xiong et al. (2021). Gene essentiality was evaluated by running an FBA biomass maximization as described above using the default bounds without an antibody constraint. A gene was considered essential if restricting the flux of all reactions that depends on it to zero caused the model to be infeasible. Genes correctly predicted as essential were classified as true positive (TP) predictions, incorrectly predicted as non‐essential were classified as false negative (FN) predictions, correctly predicted as non‐essential were classified as true negative (TN) predictions, whereas those incorrectly predicted as essential were classified as false positive (FP) predictions. The specificity and sensitivity of the model were calculated using the following equations:

$$\mathrm{Sensitivity}=\frac{\mathrm{TP}}{\mathrm{TP}+\mathrm{FN}},$$

$$\mathrm{Specificity}=\frac{\mathrm{TN}}{\mathrm{TN}+\mathrm{FP}}.$$

#### 2.4.3 Experimental work to confirm model findings

##### Cell culture

The cell line used in this study is the CHO‐GS = 46, a CHO‐K1 derived line, kindly donated by Lonza Biologics. It expresses glutamines synthetase and the cB72.3 chimeric IgG4 (immunoglobulin of γ isotype and 4 subclass) antibody. The cell line was revived from frozen cell banks and cultured at CD‐CHO medium (Thermo Fisher Scientific) addition of 1 mL/L of medium 25 mM MSX, as a selection marker. The seeding density at revival was 3 × 10^5^ and for the subsequent subcultures 2 × 10^5^ viable cells/mL. One hundred and twenty‐five milliliters Erlenmeyer flasks (Corning Inc.) with a 30 mL working volume were used to grow cells, at 140 rpm in a humidified 36.5°C incubator with 8% CO_2_ supply and were passaged every 3–4 days. The cell concentration and viability were measured using the trypan blue dye exclusion method.

##### Cell lysis

1 × 10^7^ viable cell pellets were rinsed with 4°C phosphate‐buffered saline (PBS) before cell lysis in 200 μL of RIPA Buffer (Sigma‐Aldrich) supplemented with 1% (v/v) protease inhibitor cocktail (Sigma–Aldrich). Samples were gently vortexed for 10 min before sonication on ice using three bursts of 5 s each at 25 s intervals and an amplitude power of 20%. Cell debris was removed by centrifugation at 14,000*g* for 15 min. The supernatant was stored at −80°C before western blot analysis.

##### Western blot

Sodium dodecyl sulfate polyacrylamide gel electrophoresis (SDS‐PAGE) was performed with Mini‐PROTEAN Tetra Cell (Bio‐Rad) using 12% Tris‐HCl SDS‐PAGE gels (running gel) and 5% Tris‐HCl stacking gel.

Cell lysates were diluted using a 2× SDS‐loading dye (2× SDS loading dye 0.09 M Tris‐HCl, pH 6.8; 20% glycerol; 2% SDS; 0.02% bromophenol blue; 0.1 M DTT) to a final concentration of 1× SDS‐loading dye and then with PBS to a final protein concertation of 2–10 mg/mL. All samples were denatured by heating them for 10 min at 95 °C. For loading the gel, 5 μL of each protein sample was used as well as 5 μL of the Thermo Scientific™ PageRuler™ Prestained Protein Ladder (10–180 kDa) for the identification of the band size. Gels were run at constant amplitude (25 mA/gel) for 90 min, with 1× 10xTris Glycine SDS buffer (0.25 M Tris, 1.92 M Glycine, 1% SDS, pH 8.6).

For western blot analysis a Mini Trans‐Blot® Cell was used for electroblotting the gels to an Immobilon®‐FL PVDF membrane (Millipore Ltd). The arrangement of the semi‐dry western blot involved one foam pad (Bio‐Rad), two blot filter paper (Bio‐Rad), the gel, the PVDF membrane 2 filter papers, and one foam pad. All components were previously soaked in transfer buffer (per liter: 3.03 g Tris base, 14.27 g glycine, 20% methanol). The PVDF membrane was activated as follows: the membrane was incubated in methanol for 30 s, then in deionized H_2_O for 2 min, and finally in transfer buffer for a minimum of 5 min. The gels were washed multiple times with deionized H_2_O before placing to transfer buffer. The transfer was run for 60 min at 100 V/0.35 A in ice.

For the blotting the following buffers were used: 10× TBS stock, per liter, autoclaved before use: 80 g NaCl, 2 g KCl, 30 g Tris Base, pH 8.0 with HCl), 0.05% TBS‐T (0.05% Tween‐20, 1× TBS); 0.5% TBS‐T (Washing Buffer: 0.5% Tween‐20, 1× TBS); blocking buffer (5% skimmed milk in 0.05% TBS). All the blotting procedures took place at room temperature on a shaking platform. After the transfer, the PVDF membrane was blocked in blocking buffer. An hour of primary antibody incubation (diluted in blocking buffer) followed. The primary antibody was diluted in blocking buffer according to the manufacturer's instructions. The residual primary antibody was washed off using 3× TBS‐T washing buffer for 5′. A secondary antibody (Goat Anti‐Rabbit IgG H&L from Abcam) was incubated for 30 min (1:2000 dilution in blocking buffer). The residual secondary antibody was removed with 3× TBS‐T washing buffer for 5′. The membrane was finally washed 2 × 2 min wash with deionized H_2_O. Finally, bands were developed with 5 mL of alkaline phosphate substrate BCIP/NBT kit (Thermo Fisher Scientific).

### 2.5 Data analysis and visualization

Principal component analysis (PCA) was performed on mapped predicted intracellular fluxes for each model using JMP 16 pro (SAS Institute Inc.). Visualizations and analysis were performed using Excel and JMP 16 pro (SAS Institute Inc.). Flux diagram maps were created using Escher (Rowe et al., 2018).

## 3 RESULTS AND DISCUSSION

### 3.1 Generation of the most comprehensive CHO GEM to date, iCHO2441

Since the publication of the first Chinese hamster GEM in 2016, iCHO1766 (Hefzi et al., 2016), several updates and expansions have been made to improve the model (Table 2). These include the addition of an integrated core protein secretory pathway, iCHO2048, enabling the computation of energetic cost and machinery demand of each secreted protein, as well as the creation of product‐specific GEMs (Gutierrez et al., 2020). Furthermore, iCHO1766 has been recently improved through extensive gap‐filling, un‐lumping, removal of dead‐end reactions, and updated GPR associations, resulting in the generation of iCHO2291 (Yeo et al., 2020), which is more metabolically complete than iCHO1766 but still lacks the in‐depth protein secretory machinery present in iCHO2048.

**Table 2:** Comparison of current Chinese Hamster Genome Scale models.

Model	Number of reactions	Number of genes	Gene per reaction ratio	Detailed protein secretory machinery?	Reference
iCHO1766	6663	1766	0.265	No	Hefzi et al. (2016)
iCHO2048	6764	2048	0.303	Yes	Gutierrez et al. (2020)
iCHO2101^a^	7437	2101	0.283	No	Fouladiha et al. (2021)
iCHO2291	6236	2291	0.367	No	Yeo et al. (2020)
iCHO2441	6337	2441	0.385	Yes	This work

^a^ iCHO2101 was not analyzed in this work.

As a result of these developments, there are now several Chinese hamster GEMs (Table 2). The differences among these models may impact the accuracy and reproducibility of results. Herein, we present a comprehensive Chinese hamster GEM, iCHO2441, that couples the protein secretory pathway presented in iCHO2048 (Gutierrez et al., 2020) to iCHO2291 (Yeo et al., 2020), following the methodology presented above. To our knowledge, this is one of the most complete Chinese hamster GEM to date with the highest number of genes and highest gene per reaction ratio, which makes it an ideal vehicle for CHO cell ‘omics integration.

### 3.2 Generation of cell line‐specific models

The reduction of full‐size generic GEMs via the integration of ‘omics data are a key method by which context‐ and cell line‐specific models may be generated (Gu et al., 2019; Opdam et al., 2017). These are particularly valuable since iCHO models are generic representations of Chinese hamster cells and not necessarily the CHO cell lines commonly used in industry. While cell line‐specific model generation via ‘omics integration has been widely applied to other organisms (Fang et al., 2018; Lewis et al., 2021; Nanda et al., 2020; Siriwach et al., 2020), there are limited examples of such work in CHO cells (Hefzi et al., 2016; Huang & Yoon, 2020; Schinn et al., 2021a; Yusufi et al., 2017). Cell line‐specific models can, in theory, better capture metabolic features of the cell line at hand leading to improved model predictions and therefore more accurate model‐led decision‐making, for example, for cell line and process engineering. Furthermore, it has recently been demonstrated that a small‐scale CHO cell stoichiometric model can outperform full‐size CHO cell GEMs in growth rate predictions (Antonakoudis et al., 2021). Reducing the number of reactions within the GEM may therefore bring predictive performance in line with smaller stoichiometric models by reducing how underdetermined the GEM is while still maintaining the in‐depth metabolic descriptive ability of a full GEM.

Cell line‐specific models for CHO‐S and CHO‐K1 cell lines were generated using two published transcriptomics datasets (Hefzi et al., 2016; Sumit et al., 2019) from the iCHO2441 model utilizing two non‐computationally intensive model extraction algorithms, GIMME (Becker & Palsson, 2008) and CORDA (Schultz & Qutub, 2016). iCHO2441 was selected as the parental model as it is the model with the highest number of genes so has the greatest capacity for ‘omics integration methods. As such, throughout the analysis any change in cell line‐specific model performance should be compared to the parental iCHO2441 model. The CORDA algorithm significantly reduced model size, pruning 57% of the model to 3544 reactions on average. This reduction was consistent with literature where it has been reported that the CORDA algorithm generates highly reduced metabolically functional models (Dougherty et al., 2021). Conversely, the GIMME algorithm conservatively reduced the model, pruning only 14% of the model to 5486 reactions on average. These differences in reconstruction size are largely caused by differences in expression cut‐off methodology and algorithmic differences, such as how the algorithms deal with non‐GPR‐associated reactions—GIMME heavily favors their inclusion whereas CORDA does not.

#### 3.2.1 Genome scale models can capture known CHO cell phenotypes

##### Models can predict growth rate trends across multiple cell culture phases

**Figure 1:**
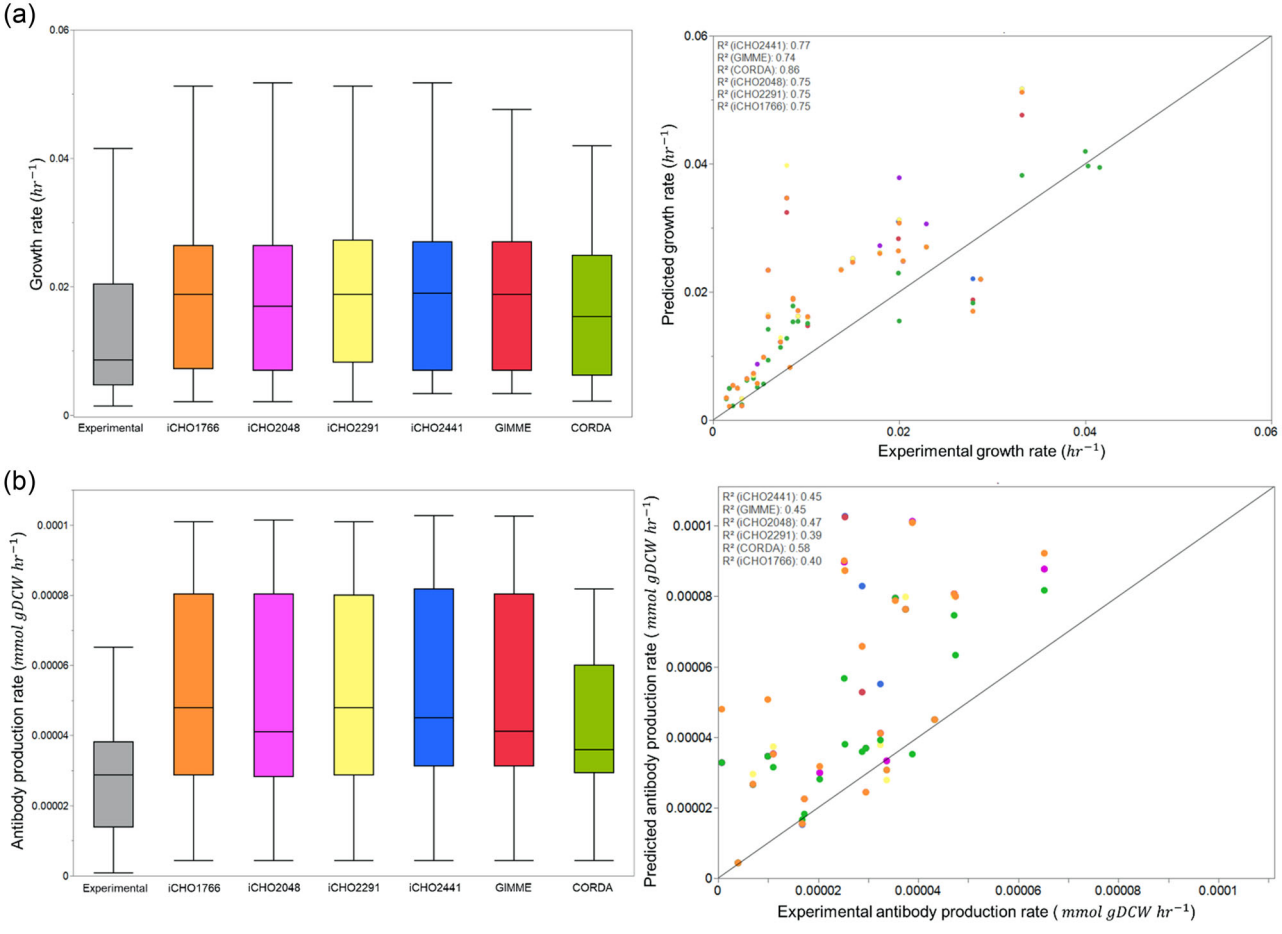
(a) Growth rate predictions across models and comparison with experimental growth rate. (b) Antibody productivity predictions across models and comparison with experimental antibody productivity.

To investigate predicted theoretical maximum growth and productivity rates for each set of models, FBA was performed either maximizing biomass (Figure 1a) or antibody secretion (Figure 1b) per experiment. Biomass maximization is commonly used as an objective function for the exponential growth stage of cell culture, whereas IgG maximization is typically used for the stationary phase of cell culture (Calmels et al., 2019). In the majority of instances, models overpredicted growth and productivity rates. This is unsurprising since the reductionistic approach of a single objective function is unlikely to be representative of complex mammalian cell lines. Despite this, qualitative trends were relatively strong (*R* ^2^ > 0.7) for biomass maximization across all models, displaying good performance in all phases of culture. Conversely, qualitative trends for IgG maximization predictions were far weaker (*R* ^2^ > 0.4), highlighting biomass maximization as a more appropriate objective function for qualitative predictions across all stages of culture where net growth is observed. All models provided comparable predictions, with secretory models (iCHO2048 and iCHO2441) providing a moderate increase in antibody production predictive performance over their non‐secretory counterparts. While this improvement was encouraging, the modest difference was surprising given the highly different protein production representations between secretory and standard models, as the higher energetic cost associated with protein secretion in iCHO2441 and iCHO2048 was expected to reduce predictions to bring them in closer line with experimentally observed values. This finding highlights that the cost of the downstream secretory pathways (glycosylation, vesicular trafficking, etc.) in secretory models is negligible compared to the cost of transcription and translation of a protein, which all GEMs capture. Despite the impressive performance of these models at qualitatively predicting growth rate, it is critical to note that maximizing a simple single reaction objective function forces intracellular flux distributions to a state that is highly unlikely to be physiologically representative in CHO cells. As a result, simple objective function maximizations should be used with caution for intracellular metabolism predictions. Instead, researchers may wish to investigate using more complex novel objective functions such as those described by Schinn et al. (2021b), or using methods that do not rely on an objective function.

##### Amino acid auxotrophy predictions provide an insight into cell line metabolism and highlight the benefits of cell line‐specific models

**Figure 2:**
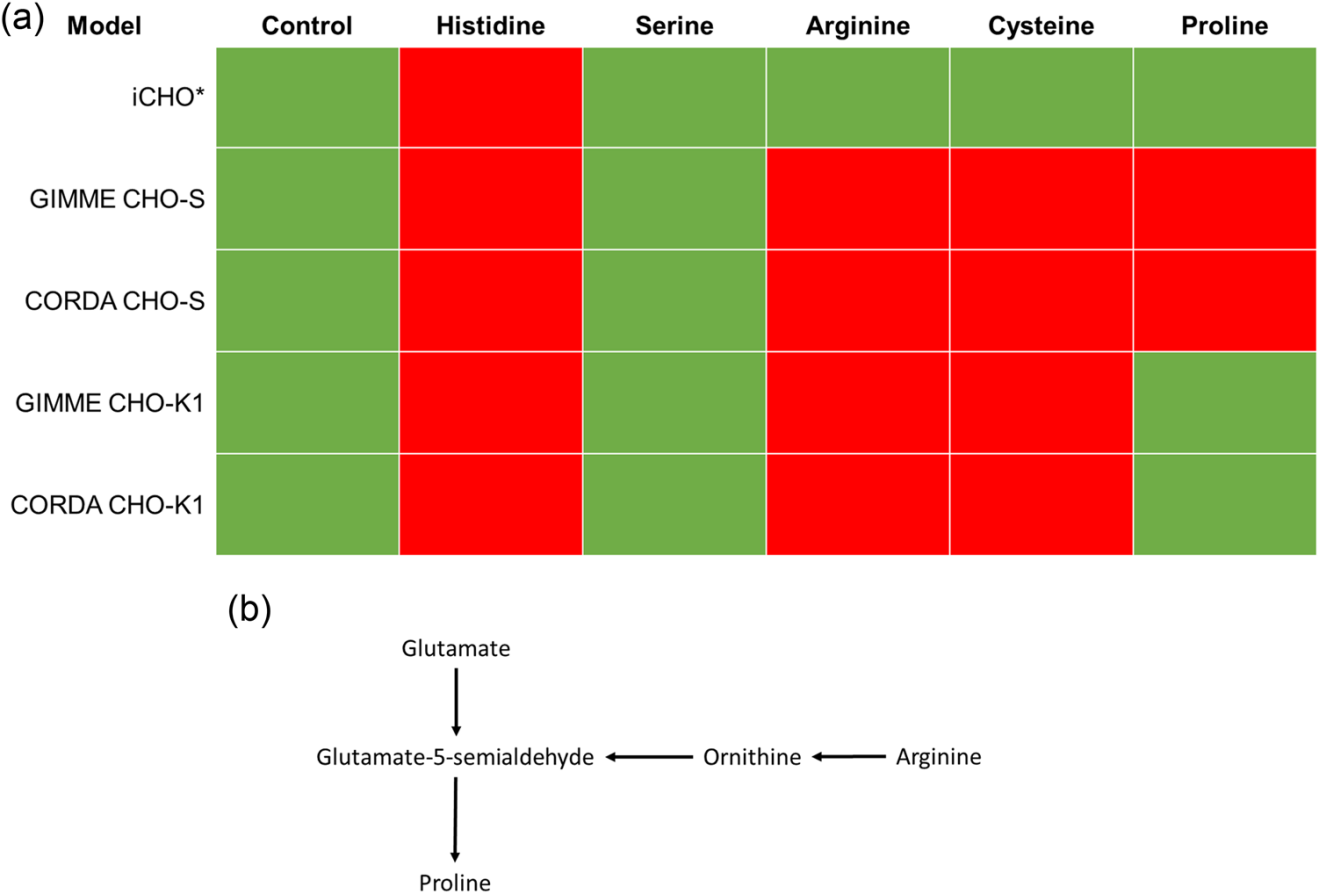
(a) Predicted amino acid auxotrophs for evaluated models. Green shading indicates viable models; red shading indicated infeasible models. (b) Proline synthesis pathway. * Indicates all full size generic iCHO model variants (iCHO1766, iCHO2048, iCHO2291, iCHO2441) display this behavior.

CHO cells are known to display several auxotrophies, including cysteine, proline, and arginine (Borman et al., 1946; Kao & Puck, 1967; Naylor et al., 1979; Valle et al., 1973) in addition to the known essential amino acids for mammalian cells (His, Ile, Leu, Lys, Met, Phe, Thr, Trp, Val). As a result, it should be possible to solve the generic iCHO GEMs in the absence of cysteine, proline, and arginine whereas cell line‐specific models should be infeasible under such conditions. We tested four generic iCHO GEMs as well as cell line‐specific models generated using GIMME and CORDA for agreement with the reported amino acid auxotrophies (Figure 2).

As anticipated, all full‐size iCHO GEMs (iCHO1766, iCHO2048, iCHO2291, iCHO2441) captured generic mammalian auxotrophies, predicting growth in the absence of arginine, cystine, and proline but not in the absence of histidine (Figure 2a). For cell line‐specific reconstructions, both CORDA and GIMME algorithms perform equivalently, accurately predicting all CHO cell auxotrophies for CHO‐S lines. This ability to accurately capture phenotypic traits such as amino acid auxotrophies is highly advantageous when using GEMs to design media formulations and feeding regimes and ultimately reinforces how important the use of such models is industrially.

Significantly, both GIMME and CORDA cell line‐specific models failed to capture the proline auxotrophy for CHO‐K1. Proline can be synthesized from arginine via ornithine or glutamate, with both pathways converging upon glutamate‐5‐semialdehyde, which ultimately is converted to proline (Figure 2b). Upon further analysis, genes for the glutamate‐5‐semialdehyde dehydrogenase and ornithine transaminase enzymes were expressed at very low levels within the CHO‐S data set and reactions were accurately removed from the model by extraction algorithms. Conversely, both enzymes were expressed at levels above the cut‐off thresholds within the CHO‐K1 data set (~1 and ~6 RPKM, respectively), resulting in GIMME maintaining glutamate to proline reactions and CORDA maintaining orthinine to proline reactions in their final reconstructions. Although expression of the glutamate‐5‐semialdehyde dehydrogenase gene in CHO‐K1 cells has previously been reported in the literature (Xu et al., 2011), proteomic work has failed to identify glutamate‐5‐semialdehyde dehydrogenase within CHO‐K1 cells (Baycin‐Hizal et al., 2012). We conduct in‐house Western blot analysis to confirm whether the enzyme was present in a CHO‐K1‐derived cell line. Specifically, we attempted detection with two commercially available antibodies that had been previously used to detect glutamate‐5‐semialdehyde dehydrogenase in mouse, rat, and human tissue. However, it was not detected in any of the samples (Supporting Information: Figure S3). This suggests that, while the gene may be transcribed, it is not being effectively translated into a functional enzyme. We further hypothesize that since aldh4a1, which converts proline to glutamate, is expressed in CHO‐K1 cells, aldh18a1, which converts glutamate to proline, may be redundant for cells grown in proline‐containing media. This highlights the issue of integrating only transcriptomic data into GEMs, as, while these model extraction algorithms can accurately capture known cell line‐specific phenotypes, they are only as accurate as the datasets and cut‐off thresholds used. Integrating multiple ‘omics datasets and using biological knowledge to manually curate outputs may result in higher quality cell line‐specific models that bridge any potential gap between transcription, translation, and reaction flux.

##### Genome‐scale models can capture experimentally validated essential genes

**Figure 3:**
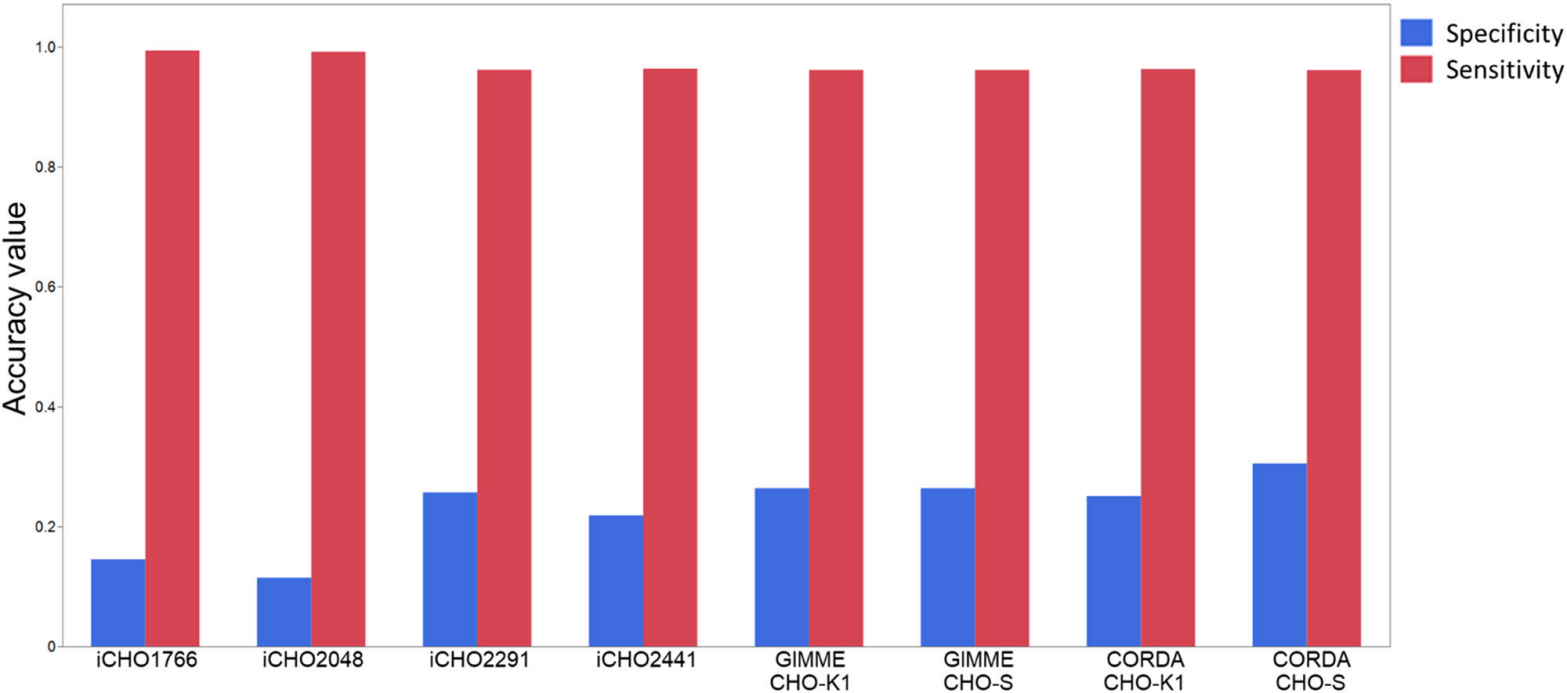
Gene specificity and sensitivity across evaluated models.

GPR associations are used to predict genes that are essential for a given task. This can act as an excellent metric for comparing how well models capture biological reality. Herein, genes predicted as essential for biomass production were evaluated and compared to an experimentally validated list of essential genes for CHO cell viability (Xiong et al., 2021) (Figure 3). The majority of models display very high specificity, rarely classifying a non‐essential gene as essential, but display lower sensitivities, failing to capture all essential genes. We hypothesize this is due to several reasons. Firstly, it is reflective of the fact that metabolic models only assess the essentiality of each gene through metabolism and cannot take into consideration other essential cellular processes such as regulatory effects. Furthermore, FBA provides a highly flexible representation of metabolism that is constrained only by the stoichiometry and reaction constraints, meaning alternate pathways to compensate for loss of function can be recruited in silico that are not possible in vivo due to phenomena such as metabolic intermediate toxicity (Price et al., 2003).

Due to its updated GPR associations and significantly higher number of genes, iCHO2291 boasted a 75% increase in specificity of prediction compared to iCHO1766. Despite predicting identical essential genes as their non‐secretory counterparts, secretory models displayed lower reported specificity. This is caused by the fact that these secretory models failed to accurately classify secretory genes as essential. This is because to reflect the nonproducing CHO clone used to experimentally validate essential genes (Xiong et al., 2021), antibody production was not constrained when running the GEMs, meaning protein production and secretion genes were predicted as non‐essential, resulting in lower prediction specificity. Notably, when antibody production was constrained, secretory models demonstrated improved specificity in line with non‐secretory models, able to capture more essential genes (data not shown). This is a key flaw of current CHO cell GEMs, as they fail to capture essentially of genes involved in protein production for cellular processes outside of antibody production. This highlights the need for the development of more advanced CHO cell models that couple metabolism with transcription and translation machinery, such as ME (metabolism and macromolecular expression) models (Thiele et al., 2009).

Significantly, all cell line‐specific models better captured essential genes compared to their parental iCHO2441 GEM. This is because cell line‐specific model extraction removes likely non‐existent alternate routes that could theoretically compensate for loss of function due to the removal of essential reactions. Interestingly, the CORDA CHO‐S model displayed a 40% increase in sensitivity compared to the 20% increase for GIMME models and 15% increase for CORDA CHO‐K1 GEM compared to the parental GEM. As the experimentally determined essential gene results were generated using a CHO‐S cell line, it is possible that the manner in which the CORDA algorithm highly reduces the parental model better captured essential genes in a biologically meaningful manner, resulting in the biggest improvement in essential gene prediction. This extraction therefore may not have been as well captured when models were not as heavily pruned or when a CHO‐K1 transcriptomics data set was used, again highlighting the importance an accurate data set and choice of extraction algorithm (and their parameters) can have on the quality of cell line specific models. The ability to predict essential genes is indispensable when using GEMs to design cell engineering strategies or identifying potential selection markers. The use of cell line‐specific models over generic GEMs is therefore highly recommended for these tasks.

### 3.3 CHO cell metabolic models predict trends in intracellular fluxes

#### 3.3.1 Flux sampling is the preferable method to assess intracellular flux predictions

The ability to accurately predict intracellular metabolic fluxes is vital if researchers wish to utilize metabolic models for performance attribute identification and design of engineering strategies. Moreover, measuring how accurately metabolic models can capture intracellular fluxes is an excellent measure of model performance, allowing models to be scrutinized and compared. Despite this, there is limited robust evidence assessing how well CHO cell metabolic models are able to predict intracellular fluxes. Herein, flux sampling was utilized to assess each model's intracellular predictive capacity, which is a distinct method from, and has several advantages over, the more prevalent FBA technique. FBA is an optimization methodology, which maximizes or minimizes a cellular objective function subject to constraints. In most cases, the optimal solution presented by FBA can be achieved by an infinite combination of intracellular fluxes. This is due to the mathematical nature of flux analysis using GEM, where the number of unknown variables (reaction fluxes) will always exceed the number of equations (material balances) and experimental measurements, causing large degrees of freedom. For this reason, the intracellular fluxes predicted by FBA are a particular set of feasible reaction fluxes that satisfy the particular optimality criterion. This means that FBA alone is not suitable for understanding the model's ability to predict reaction fluxes that are not specified in the objective function. Another challenge is the requirement for an objective function. The use of an objective function implies that the cell is trying to maximize or minimize a particular cellular function, which, as previously discussed above, is not true in vivo.

To truly capture intracellular fluxes therefore, the entire feasible solution space must be studied, which can be achieved through flux sampling. Flux sampling uniformly samples the feasible solution space many times, producing an indication of the likely distribution of all fluxes. This allows for effective comparison with experimental reaction fluxes. Flux variability analysis (FVA) (Gudmundsson & Thiele, 2010) is another technique which can show the feasible range of fluxes, but this method is incapable of determining the probability distribution of fluxes. Flux sampling has the additional advantage in that no objective function is required, only reaction constraints, allowing fluxes to be analyzed without the assumption of a cellular objective.

For this analysis we constrained the models using experimental metabolite uptakes, cell growth and recombinant protein productivity from datasets in Supporting Information: Table S2. These types of constraints are the most frequently encountered in flux analysis, as they are routinely measured and easy to implement into metabolic models. The models presented here can be considered as “base‐case” version of the models, because they constrain with basic extracellular metabolomic, antibody secretion and growth rate data only. As a result, future work assessing model improvement methods may use these results as a control comparison to assess if any improvement in predictive performance has been achieved.

To assess model capacity to predict intracellular flux distributions, mean predicted flux sample values are compared with ^13^C determined intracellular flux values for each reaction across the 31 experiments, from studies in Table 1. Significantly, it is important to note that while ^13^C fluxes are likely to be accurate, they are determined using MFA and are therefore influenced by the metabolic network used and inherently involve a level of prediction. As a result, ^13^C fluxes should not be seen as experimental values, rather as gold standard predictions.

#### 3.3.2 CHO cell models are effective at predicting overall trends in metabolism

The initial analysis demonstrated that the secretory models (iCHO2048 and iCHO2441) performed comparably to their nonsecretory counterparts (iCHO1766 and iCHO2291), with highly similar flux distributions between iCHO1766/iCHO2048 and iCHO2291/iCHO2441 (Supporting Information: Figure 4) and no significant difference in model performance metrics. This demonstrates that the addition of the secretory machinery to the models does not significantly impact intracellular flux predictions when running models using “base‐case” methodology, with any difference in performance being due to the core metabolic map. As a result of this similarity, only in‐depth analysis of iCHO1766 and iCHO2441 is presented here but their results can be seen to be analogous to iCHO2048 and iCHO2291 respectively.

**Figure 4:**
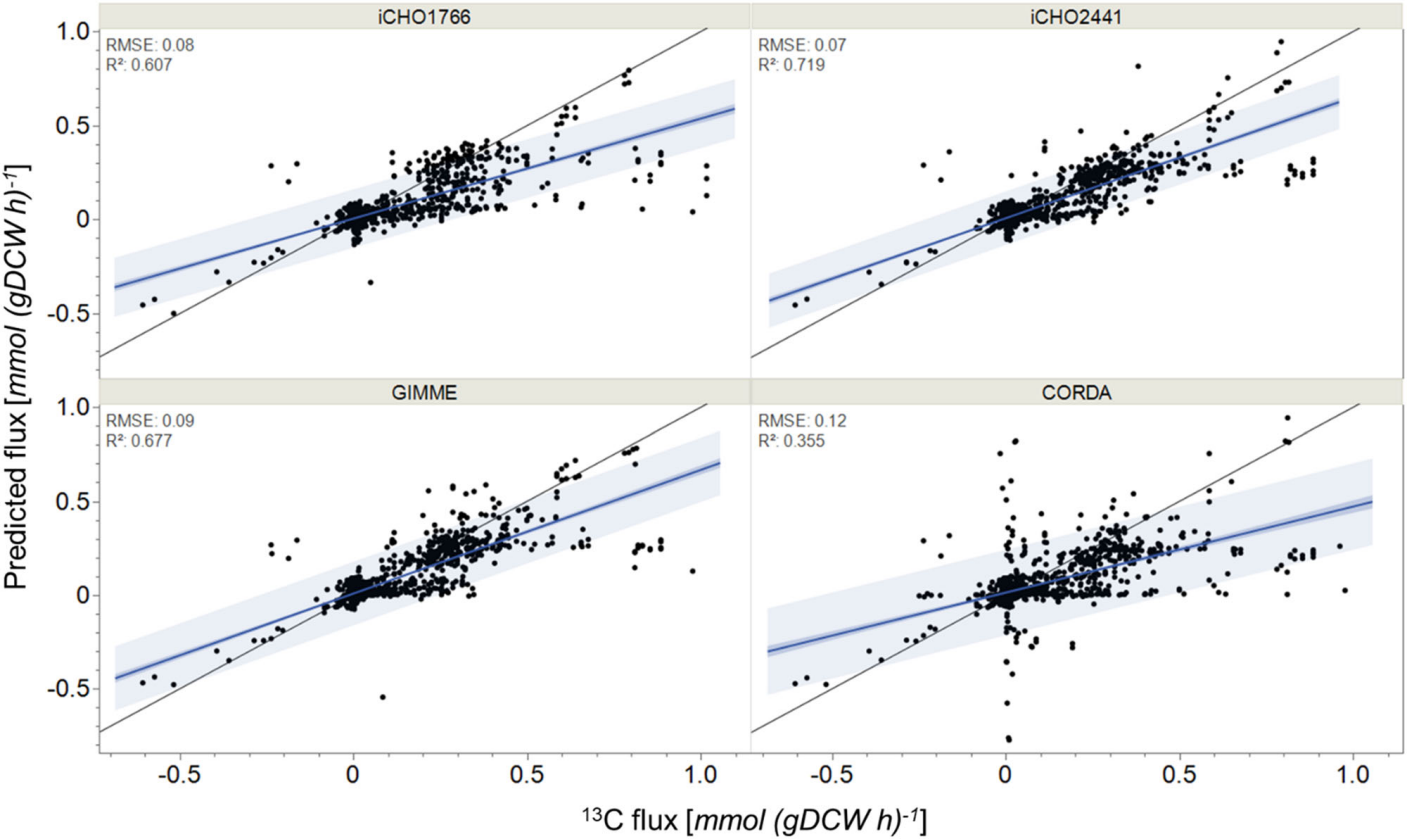
Predicted mean flux samples against ^13^C‐labeled experimental fluxes.

To evaluate model capacity to predict broad intracellular flux distributions, mean predicted flux sample values were plotted against ^13^C determined intracellular flux values. All models successfully captured broad metabolic trends to varying degrees (Figure 4). This was particularly impressive, given this was a “base case” assessment of model performance, with no advanced constraining methods or assumed cellular objectives were applied. Interestingly, the majority of reactions were underpredicted for all models, sitting below the line of equality. In particular, models struggled to predict reactions which displayed experimentally reported flux >0.6 mmol gDC W^−1^ h^−1^. This underprediction of fluxes is potentially caused by several factors. Firstly, it may be a result of the use of a generic biomass equation, which did not effectively capture the true biomass composition of cells resulting in an underprediction of fluxes. It may also be due to the lack of an ATP maintenance constraint, which has been shown to improve intracellular flux predictions (Széliová, Štor, et al., 2020). Finally, it may be indicative of the “metabolic efficiency” of these models compared to in vivo metabolism, meaning lower flux values through core reactions are able to satisfy the model constraints. This would therefore result in the key set of reactions that are experimentally measured being underpredicted, while the remainder of flux is able to move into peripheral reactions within the models meaning, arguably, non‐^13^C determined values of peripheral metabolism may be overpredicted within the models. This ultimately highlights how important it is to accurately predict central metabolism, as accurately predicting central reaction fluxes increases the likelihood of improved predictions of peripheral metabolism, for example, fatty acid metabolism.

#### 3.3.3 Model choice impacts intracellular predictive capability across different reactions

As well as comparing broad flux predictions between CHO cell models, the in‐depth analysis of predictive performance of individual intracellular reactions and pathways can facilitate a deeper understanding of these pathways and the CHO cell models themselves. This insight is key when using intracellular flux predictions for real‐world applications, such as identifying genetic engineering targets. The confidence in intracellular flux predictions must be quantified and understood to achieve a comprehensive analysis.

To compare model performance, the ability of each model to predict reactions both qualitatively and quantitively was interrogated. Qualitative predictive power was defined as the ability to predict trends in intracellular reactions between experiments, through the Pearson correlation coefficient, ρ. Correlation evaluates if the model can understand a differential change in flux between two conditions, regardless of scale. This is important, as picking up qualitative changes in metabolism is extremely useful to examine cell metabolism, regardless of the proximity to the experimental flux value.

Quantitative predictive power is defined by a new metric introduced in this paper, named Capability. Capability is the percentage of flux samples that are correctly predicted to be within the ^13^C range for a given reaction. A high Capability means that the model can *accurately* predict a given reaction, as many of the predicted values fall within the experimental range. As we are dealing with base‐case analysis in this paper, many of the fluxes sampled have large ranges, resulting in only a small percentage of fluxes being correct. This means that the model could predict the correct value if it is guided toward this solution using a more advanced flux analysis technique. Significantly, Capability helps to mitigate any uncertainty regarding ^13^C flux values by the use of upper and lower flux bounds and not a fixed value.

**Figure 6:**
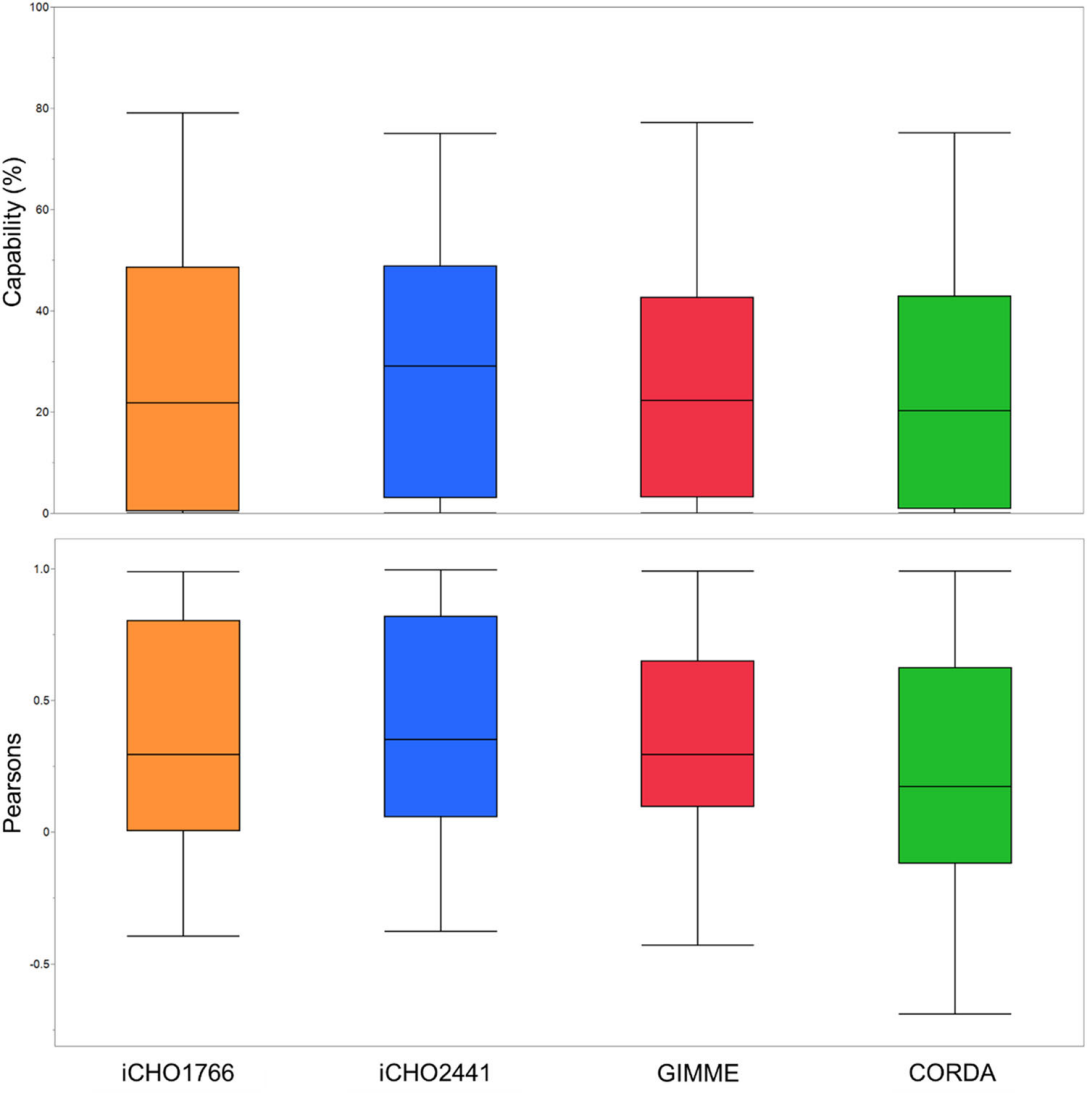
Box plot of Capability and Pearson correlation for evaluated models.

The highest performing model was iCHO2441, the updated GEM presented in this work. iCHO2441 is a marked improvement upon iCHO1766 in its ability to capture broad flux distributions (Figure 4), qualitative trends in reaction fluxes (Figure 5a and Figure 6), and to predict experimental fluxes (Figure 5b and Figure 6). Significantly, despite better capturing extracellular traits of CHO cell lines, cell line‐specific GEMs did not improve intracellular flux predictions compared to the generic iCHO2441 GEM. Due to the relative similarities between GIMME and iCHO2441, there was little difference on average between predictive performance of the two models, with GIMME‐generated cell line‐specific models performing slightly worse. CORDA‐generated cell line‐specific models performed worse again, displaying lower average ρ and Capability than both iCHO2441 and GIMME, despite containing a significantly reduced number of reactions. Arguably, this reinforces previous findings about the gap between transcription, translation, and reaction flux, emphasizing the need to integrate multiple ‘omics sources GEMs. Given the difference between the performance of the GIMME and CORDA model extraction approaches it is expected other model extraction algorithms and parameters would also give differing performance, especially given GIMME has previously been shown to have weaker performance than other extraction methodologies (Jamialahmadi et al., 2019). As such, trailing these alternate solutions is advisable when constructing cell line‐specific algorithms. While such model extraction method analysis is outside the scope of this work, there is extensive relevant literature (Opdam et al., 2017; Richelle et al., 2019; Walakira et al., 2021) and future work may wish to expand these analyses to include intracellular flux prediction analysis. Significantly, it is worth noting that the transcriptomic data utilized here to generate cell line‐specific models were generated independently to the ^13^C datasets. This means it may not be truly reflective of cell lines used during the ^13^C experiment, which may explain the surprisingly poor predictive performance from cell line‐specific GEMs. To our knowledge, there is currently no data set available for CHO cells consisting of both transcriptomic and ^13^C data, which would allow for a more accurate assessment of how model extraction algorithms affect intracellular flux predictions. Taken together, the results presented here suggest that conservatively pruning GEMs to generate cell line‐specific models using techniques such as GIMME may provide the best trade‐off in terms of extracellular and intracellular predictive performance as it is able to better capture extracellular performance without having a significant reduction in intracellular predictive performance.

Figure 5(a) Capability and (b) Pearson correlation for each ^13^C labeled reaction for evaluated models.

**Figure:**
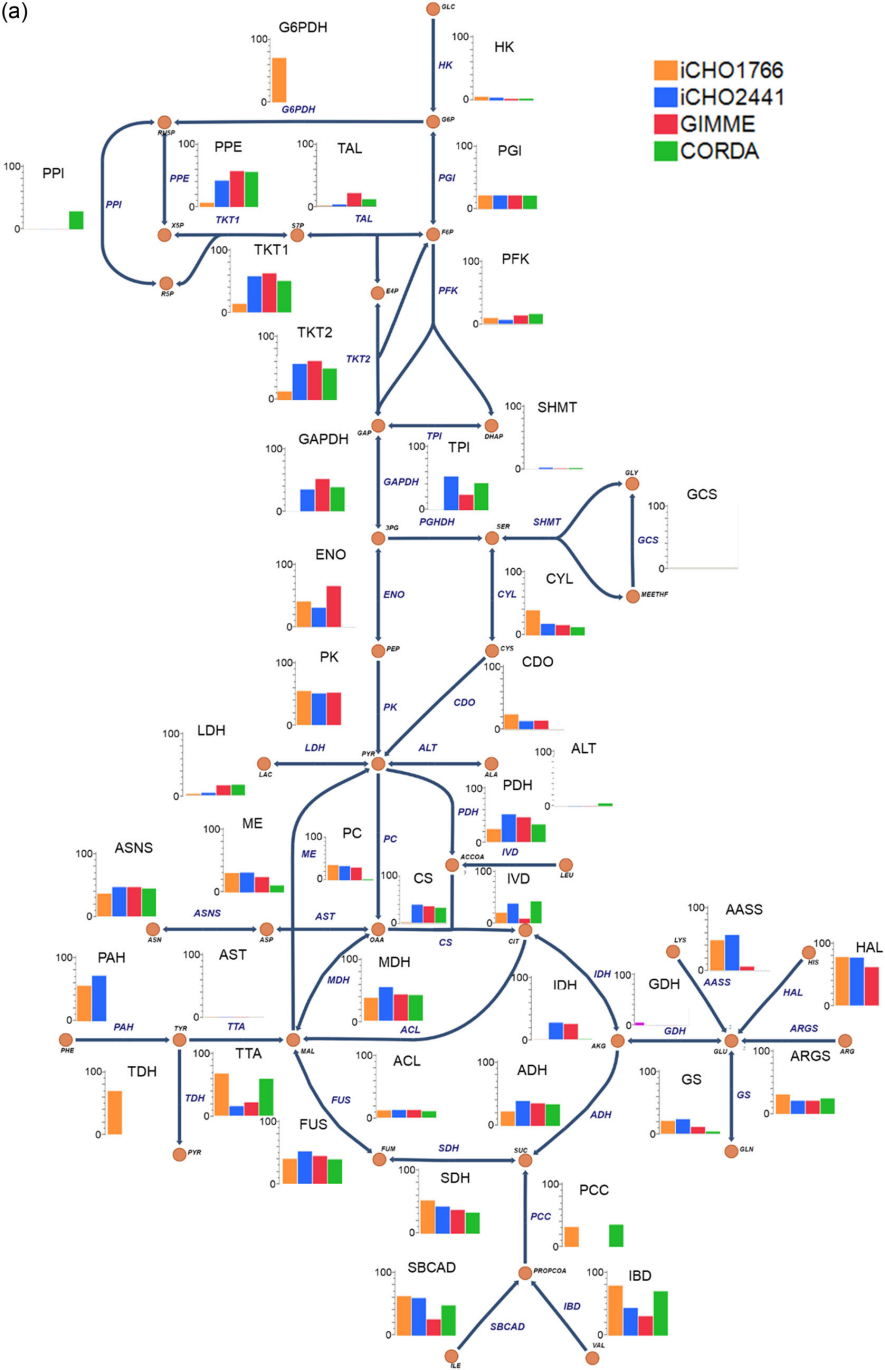

**Figure:**
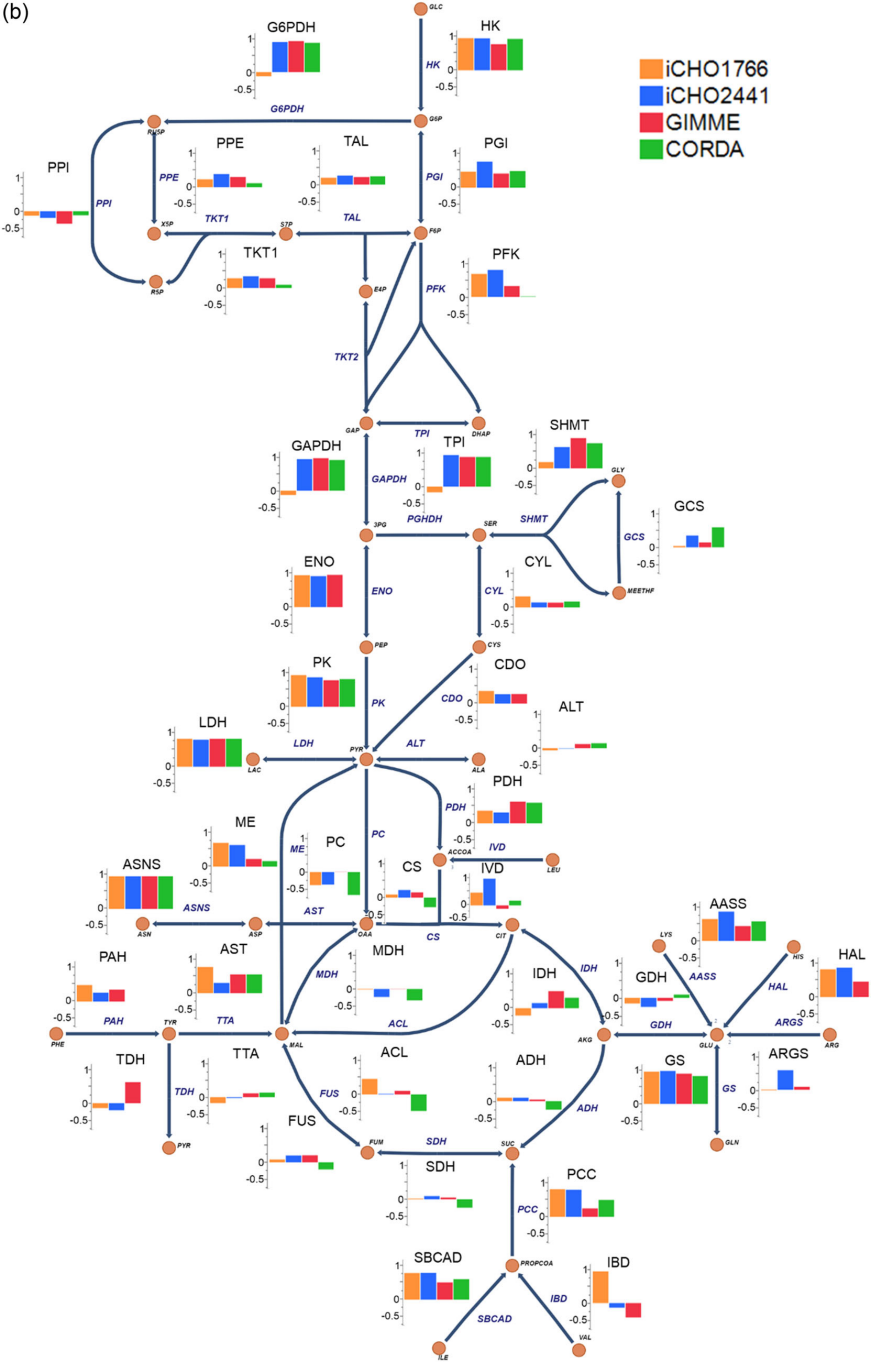

#### 3.3.4 Alternative pathways may impede predictive performance

Alternative pathways are both an asset and a hindrance to metabolic models. On the one hand, they represent the flexibility of cell biology, and any well‐annotated GEM should contain all possible pathways available to the cell. On the other hand, they introduce variability and uncertainty into flux analysis as metabolic models will allocate flux to alternative pathways if they are able to, which may divert flux away from biologically expected pathways (Figure 6).

An example of this is glycolysis, alongside the typical upper glycolytic reactions, full‐scale GEMs possess alternative pathways to convert glucose into glyceraldehyde 3‐phosphate (GAP) and dihydroxyacetone phosphate (DHAP). One of these is the polyol pathway, represented by reactions RE1342C and SBTD_D2, which convert glucose to fructose via sorbitol, bypassing the HEX1 and PGI reactions. As demonstrated in Supporting Information: Figure S2, both iCHO1766 and iCHO2441 consistently predicted high levels of flux for reactions RE1342C and SBTD_D2. This phenotype is associated with the pathophysiology of diabetes resulting from the saturation of HEX1 due to very high glucose concentrations (Chung et al., 2003; Lorenzi, 2007) and it is deemed unlikely to be physiologically relevant in this context.

The fructose formed from the polyol pathway is subsequently either phosphorylated to re‐enter glycolysis (HEX7) or can go through yet another alternative pathway (KHK and FBA2), bypassing the PFK and FBA reactions. The diversion of flux away from glycolytic reactions brings about an underprediction of flux and hence the poor C seen in upper glycolysis for iCHO1766 and iCHO2441 (Figure 5a). Cell line‐specific models also display low Capability in glycolytic reactions. One of the objectives of a cell line‐specific metabolic model is to remove pathways that have low expression and are not expected to carry flux in vivo. However, the enzymes involved in the polyol pathway are also involved in other intracellular reactions, such as akr1a1 (alcohol dehydrogenase) and their expression is larger than the cut‐off point for the model extraction algorithms. There is expected to be competition for these promiscuous enzymes and the low substrate availability is likely to cause low fluxes in these pathways. These physiological limitations are not captured by simple applications of GEMs, which can only consider stoichiometric mass balances as an impediment to reaction rates, which is ultimately one of the reasons why GEMs are more “metabolically efficient” than real cell cultures, as seen in Figure 4.

#### 3.3.5 Internal cycles remain a weak point for GEMs

Internal cycles, also known as thermodynamically infeasible loops or Type III loops, are a well‐studied issue in GEM analysis (de Martino et al., 2013; Desouki et al., 2015; Palsson et al., 2002; Schellenberger et al., 2011; Schilling et al., 2000; Schinn et al., 2021b; Wright & Wagner, 2008). They exist when a set of reactions have no net production/consumption of metabolites, allowing the reactions involved to take any value and still produce a feasible flux distribution.

iCHO1766 contains several internal cycles, which cause arbitrary fluxes in the cycle reactions, causing poor correlation and accuracy with experimental fluxes. Eleven of the 49 reactions had flux predictions heavily affected by internal cycles, causing low correlation and Capability. iCHO2441, on the other hand, contains fewer of these internal cycles and can predict more reasonable flux distributions. Many of these cycles present in iCHO1766 were identified and removed as part of the update procedure in the creation of iCHO2291, the non‐secretory component of iCHO2441. Internal loops in iCHO2241 affect four reactions (PPI, GDH, ALT, AST), a large improvement over iCHO1766. However, while iCHO2441 can predict these fluxes at more reasonable levels the correlation coefficients for these reactions (TPI, GAPDH, G6PDH, IDH, SHMT, GCS, PCC, PGHDH) are still poor, meaning that iCHO2441 is not able to pick up qualitative trends in the reaction rates. This suggests that the reactions involved in internal cycles are still a weak point for current metabolic models, even if the mathematical inconsistency is removed, and must be a target for future improvements.

## 4 CONCLUSIONS AND OUTLOOK

Here we present an evaluation of extracellular and intracellular predictive power of CHO cell genome‐scale models using a holistic ^13^C‐labeled fluxomic assessment methodology. Firstly, we combined the metabolic networks of iCHO2291 and iCHO2048 to generate a new CHO GEM for the community to employ, iCHO2441, and from this, generated industrially relevant CHO‐K1 and CHO‐S cell line‐specific models. We demonstrated that CHO GEMs have good performance at extracellular phenotypic prediction, being able to understand qualitative trends in growth rate predictions across many different culture conditions. This gives the community confidence in robustness and broad metabolic applicability of CHO cell GEMs. Cell line‐specific models are additionally able to capture known cell auxotrophies and essential genes, indicating they have an advantage in predicting some biologically relevant flux distributions. However, they are shown to have a moderate decrease in intracellular prediction performance over full‐scale models and suggest that industry should consider using a variety of models dependent on the industrial application. We warn of the issues in using a single transcriptomic data set for model extraction and recommend trialing a selection of model extraction algorithms and manual curation approaches to create specialized cell line models.

Secondly, we demonstrate that CHO GEMs are adept at appraising intracellular flux states across varying cell culture conditions. CHO cell GEMs can predict qualitatively and quantitatively for many intracellular reactions using only simple metabolomic datasets to inform models. This gives assurance that CHO GEMs can be used as an effective toolbox for interrogating cellular metabolism in many industrial settings. We suggest flux sampling as the preferred technique to probe intracellular flux predictions, as it can capture the entire feasible solution space and does not require the assumption of a metabolic objective. Additionally, we utilize a new metric termed Capability, which finds the percentage of intracellular flux samples falling within the correct experimental range, as a novel method to assess metabolic models.

While these base‐case CHO GEMs are adept tools, they still suffer from the presence of alternative pathways, where flux is diverted away from physiologically relevant pathways, and internal cycles, which cause arbitrary and large fluxes. Throughout this work we highlight that inaccurate intracellular flux predictions may arise from several sources. These include using poorly performing model reduction algorithms, issues with using C13 data for comparison due to its reliance on a core metabolic model for flux computation, as well as general poor performance due to the large number of degrees of freedom GEMs possess. We suggest that future work should focus on guiding flux toward biologically relevant pathways using advanced constraining methods and/or cellular objectives. This will likely involve integration of various layers of ‘omics data as well as known biological insights.

Through this analysis, we demonstrate iCHO2441 to have equivalent or improved predictive performance over the original CHO GEM, iCHO1766. While many of these improvements, particularly the reported improved intracellular predictive performance, are thanks to the impressive updates made with the development of iCHO2291 (Yeo et al., 2020), iCHO2441 maintains all the strengths of iCHO2291 while having an improved number of genes and an in‐depth description of protein secretion. This makes iCHO2441 better suited for genetic and process engineering strategy identification than previous CHO cell GEMs. This is thanks to its ability to predict central metabolism and represent protein secretion more accurately, while being able to integrate a larger amount of experimental ‘omics data, helping better exploit these rich data sources. Looking forward to the next generation of CHO cell GEMs, researchers may be able to further improve model quality by utilizing new and improved human GEMs as a backbone, such as Human1 (Robinson et al., 2020), as well as employing information in iCHO2441 and other recently updated CHO cell GEMs, such as iCHO2101 (Fouladiha et al., 2021). Taking information from these multiple sources should ensure the highest quality model possible and shall further advance the field of CHO cell genome‐scale modeling.

Overall, we have introduced an updated CHO GEM to the community and have employed the most intensive CHO cell model assessment to‐date to evaluate the predictive performance of CHO cell GEMs. We have demonstrated that CHO cell models are able to predict biological phenomena and are therefore valuable tools for understanding cellular metabolism in many industrial applications. Ultimately, this work lays a foundation for the development and assessment of next‐generation flux analysis techniques.

## Supporting information

Supporting information.

Supporting Information.

## Data Availability

The data that support the findings of this study are openly available in Mendeley Data at https://data.mendeley.com/datasets/73cmrfk8x9, reference number DOI:10.17632/73cmrfk8x9.1.
