# Supplementary material for: How reliable are Chinese hamster ovary (CHO) cell genome‐scale metabolic models?
Source: Biotechnol Bioeng. 2023 Mar 18;120(9):2460–78. doi: 10.1002/bit.28366 (PMC10952175; doi:10.1002/bit.28366)
Supplement: Supplementary file 1 — Supporting information. [file BIT-120-2460-s002.docx]

# zFPKM for CORDA gene classification


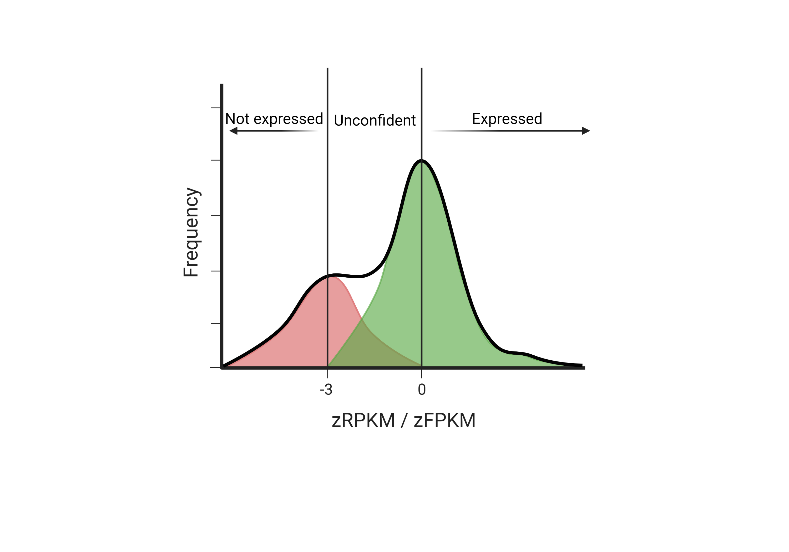


Figure S1. Gene classification strategy for CORDA using zFPKM

To date within the literature, GIMME is the main algorithm to have been used to generate CHO cell line specific models. In brief, GIMME takes ‘omics expression inputs mapped to reactions, a metabolic reconstruction, and required metabolic functionalities. A reconstruction is mapped through an omics dataset, removing reactions that are below a specified threshold, creating a reduced model. Reactions are then reinserted into the reduced model as needed using linear programming to achieve cellular viability defined as a fraction of the objective function. In order for these models to act as a ‘standard’, when using GIMME, we utilised the same expression threshold and optimality fraction parameters employed in previous studies (see material and methods).

The CORDA algorithm represents one of the most recently published model extraction algorithms and has yet to be applied to the CHO GeM. In brief, CORDA takes user-defined high, medium, and negative confidence reactions to produce a model that is consistent (i.e., all reactions can carry flux) while maximizing high and medium confidence reactions and minimizing the number of negative confidence reactions. This is achieved using a dependency assessment, where negative conﬁdence reactions, are assigned an arbitrarily high cost. This cost is then minimized while enforcing a small ﬂux through medium or high conﬁdence reactions to distinguish which negative confidence reactions are beneﬁcial for high confidence reactions to carry ﬂux that should be included in the final reconstruction.

In order to classify reactions into high, medium and negative confidence classes for CORDA, a novel approach was developed by utilising zFPKM transformation (Hart et al., 2013) to identify expressed and unexpressed transcripts. This approach overcomes the issue of non-comparability between FPKM/RPKM data across samples, and the subsequent problematic nature of setting arbitrary expressed/not expressed cut off values, by transforming the data so a consistent cut off value can be used. As demonstrated in supplementary figure S1, log transformed FPKM/RPKM RNA-Seq values generally follow a bimodal distribution of high and low expression genes (Hebenstreit et al., 2011), in which the main rightward Gaussian curve corresponds to genes that are expressed and the leftward smaller peak corresponds to leaky expression, off-target read mapping or sequencing errors. As zFPKM transformation centres the main peak over 0 it becomes possible to consistently classify genes as expressed across datasets with reasonably confidence, whereby transformed scores >0 are expressed (high confidence) and scores <-3 are unexpressed (negative confidence). We further classified genes with zFPKM scores between -3 and -1.5 and-1.5 and 0 as ‘low confidence expressed’ and ‘medium confidence expressed’ respectively. Currently, the CORDA algorithm lumps low and medium confidence reactions into the same medium confidence category. As such, future work may wish to expand the CORDA algorithm to preferentially include medium confidence reactions over low confidence reaction to improve algorithm outputs. To our knowledge this is the first time this transformation technique has been coupled to the CORDA algorithm for confidence classifications.

# Alternate pathways


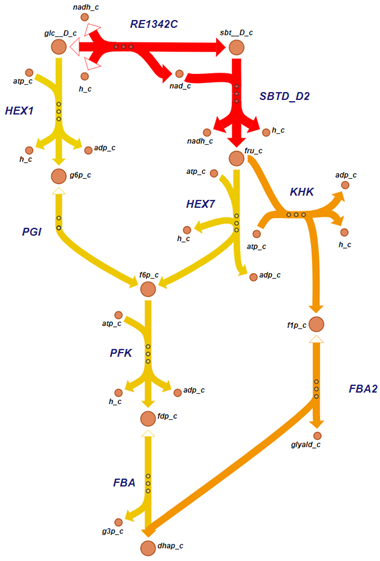


Figure S2. Glycolysis and alternate pathways in iCHO1766. Darker colour and arrow width indicate amount of flux.

# Glutamate-5-semialdehyde dehydrogenase (ALDH18A1) western blot


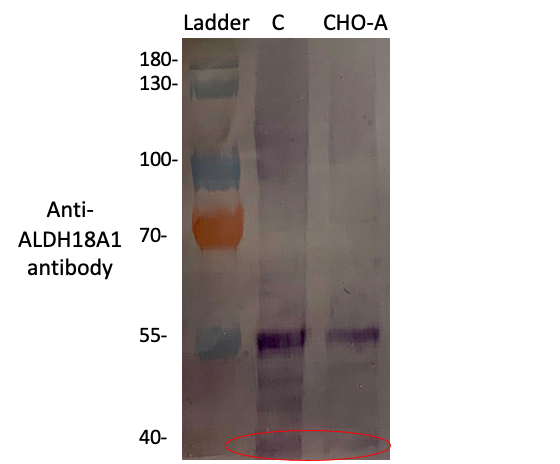

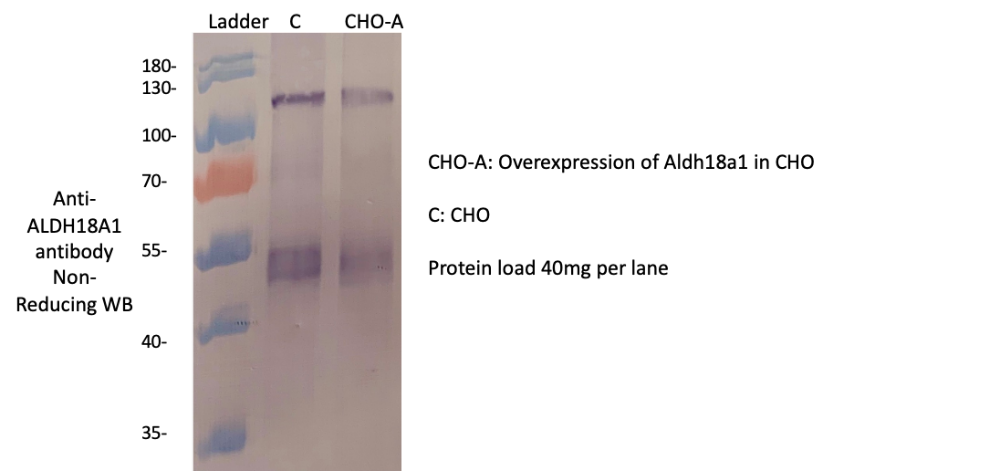


**A**

**B**


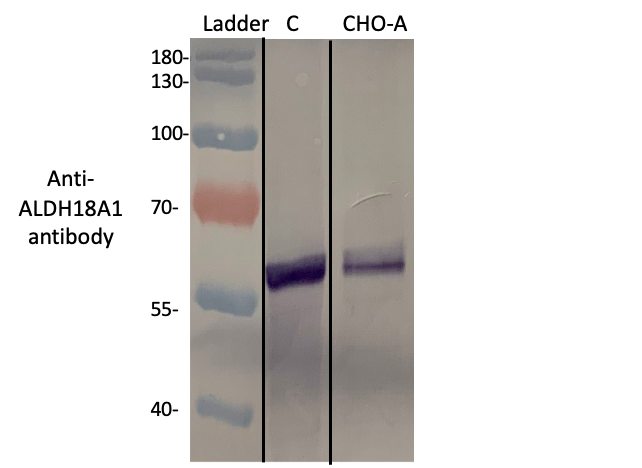


**C**

Figure S3. A) Non-reducing western blot analysis of the CHO-AA lysates, compared to the control CHO cell lysate. The antibody used is the ab206682 from abcam. B) Western blot analysis of the CHO-AA lysates, which are compared to the control CHO cell. The antibody used is the ab206682 from abcam. C) Western blot analysis of the CHO-AA lysates, compared to the control, original CHO cells. The antibody used is the ab223713 from abcam. The antibody detects the protein ALDH18A1 and has been verified in human, rat, and mouse tissue cells. However, it was not able to detect the protein in any of the samples, as the molecular weight of ALDH18A1 is around 87 kDa, and the band is at 55 kDa, much smaller than the required weight.

# Further analysis of iCHO intracellular flux distributions

Principle component analysis (PCA) of model fluxes shows clustering of iCHO1766 with iCHO2048 and iCHO2291 with iCHO2441, demonstrating that secretory models have highly similar flux distributions to their non-secretory counterparts and that it is predominantly the core metabolic map causing differences between models. Any minor differences in performance between model pairs is likely due to slight variations arising from the flux sampling methodology.


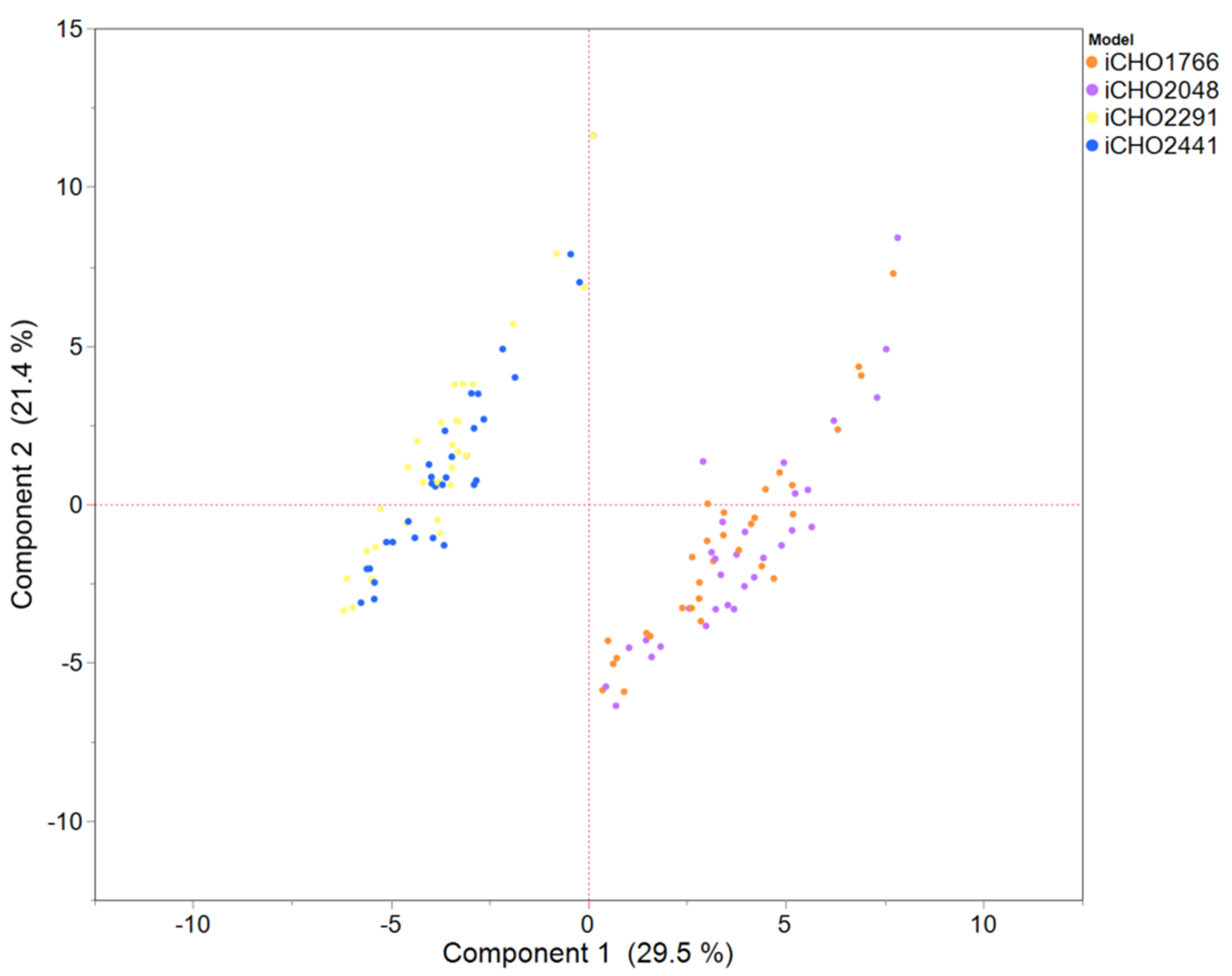


**C**

**A**

**B**


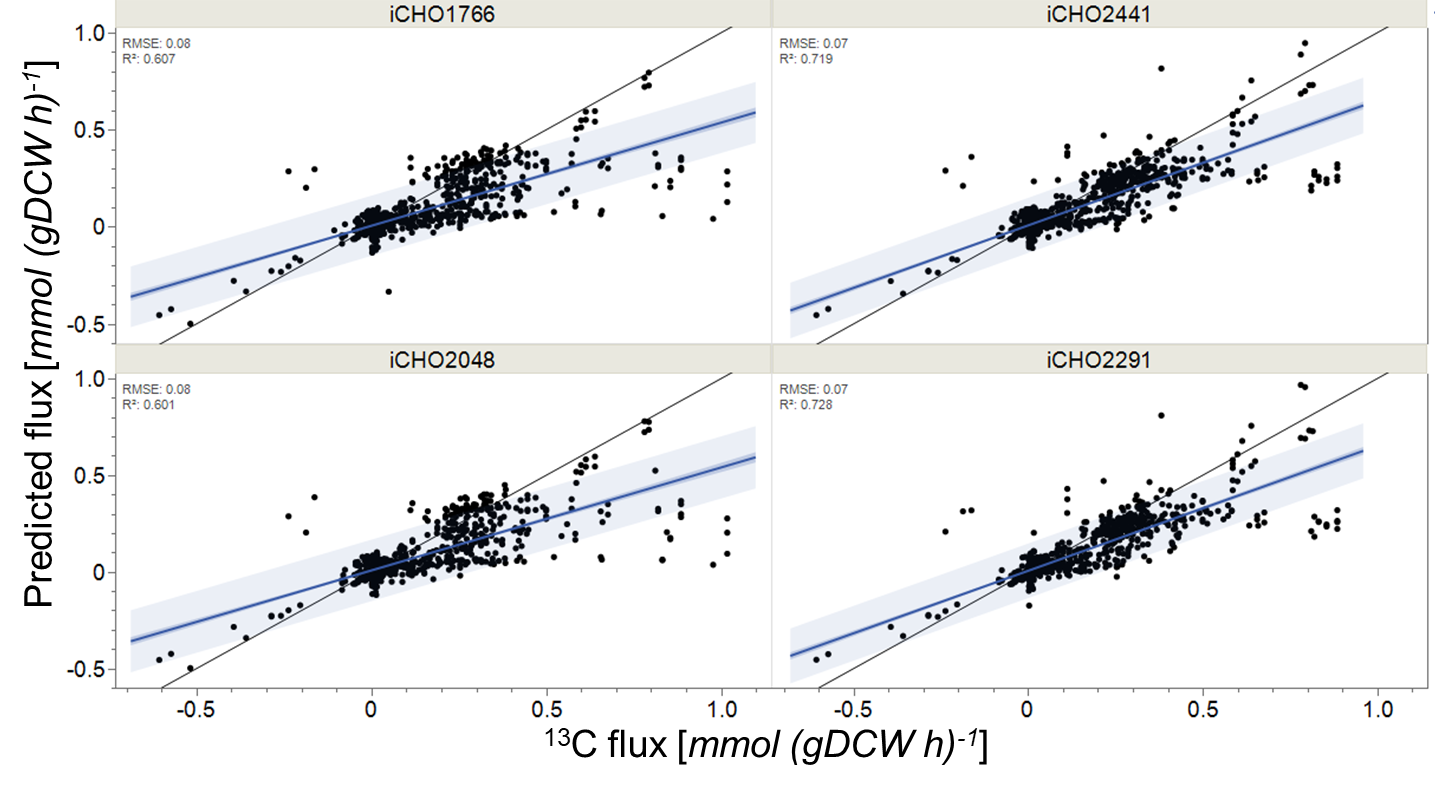


**D**

Figure S4.A) Principal component analysis (PCA) of model fluxes. B) Per reaction capability, C) Per reaction Pearsons correlation and D) Over all Predicted mean flux samples against 13C labelled experimental fluxes of iCHO models.
